# Novel laser model of optic nerve transection provides valuable insights about the dynamics of optic nerve regeneration

**DOI:** 10.21203/rs.3.rs-5085599/v1

**Published:** 2024-11-02

**Authors:** Chloe Moulin, Galina Dvoriantchikova, Niloufar Bineshfar, Ben Swingle, Gaby Martinez, Daniel Groso, Michelle Zhang, Dmitry Ivanov, Daniel Pelaez

**Affiliations:** Bascom Palmer Eye Institute, University of Miami Miller School of Medicine; Bascom Palmer Eye Institute, University of Miami Miller School of Medicine; Bascom Palmer Eye Institute, University of Miami Miller School of Medicine; Bascom Palmer Eye Institute, University of Miami Miller School of Medicine; Bascom Palmer Eye Institute, University of Miami Miller School of Medicine; Bascom Palmer Eye Institute, University of Miami Miller School of Medicine; Bascom Palmer Eye Institute, University of Miami Miller School of Medicine; Bascom Palmer Eye Institute, University of Miami Miller School of Medicine; Bascom Palmer Eye Institute, University of Miami Miller School of Medicine

## Abstract

Optic nerve (ON) injury causes blindness in adult mammals as their retinal ganglion cells (RGCs) cannot regenerate axons. However, amphibian RGC axons do not experience the same regenerative failure. Studying the regeneration process of the ON in amphibians holds profound implications for regenerative medicine and human health. Using transgenic tadpoles and laser micro-optics, we developed a reproducible ON transection and regeneration model. Through microscopy, functional testing, TUNEL, EdU assays, and RNA-seq, we characterized the ON injury response and recovery. Our model suggests no chemoattractant gradient exists early in regeneration, with defasciculated axons sprouting in random directions from the globe-proximal cut end. Once individual axons reach the appropriate anatomical insertion point in the brain, their tract is reinforced by other regenerating axons, restoring normal ON morphology. Thus, guidance cues or scaffolding from brain-innervating axons likely support later stages of regeneration. After 14 days, the regenerated ON is morphologically indistinguishable from the naïve ON, and visual function is restored. We found no evidence of RGC death or new RGC formation in the model, suggesting that only pre-existing RGCs are involved in ON regeneration.

## Introduction

Visual information is focused onto the retina, where it is captured as light impulses by photoreceptors at the posterior retina, transmitted to bipolar cells, and then to the retinal ganglion cells (RGCs) at the inner retinal layer [1]. That visual information is transmitted from the eye to the brain by the RGC axons that form the optic nerve [1, 2]. RGCs and their axons are particularly vulnerable to damage in various optic neuropathies, including traumatic optic neuropathy [1–3]. The survival of RGCs is crucial for vision maintenance since RGCs do not regenerate in adult mammals and their loss leads to irreversible visual impairment or complete vision loss [1]. Additionally, even the mammalian RGCs that survive optic nerve damage are unable to regenerate axons and restore contact with the appropriate visual targets in the brain [2, 4]. Amphibians and teleost fish, such as zebrafish, are free from these shortcomings. Thus, understanding the molecular processes of optic nerve regeneration in amphibians and teleost fish can inform the understanding or regenerative failure in the mammalian optic nerve.

Amphibians and teleost fish retain significant regenerative abilities in the limb, tail, spinal cord, and parts of the eye, including RGC bodies and axons [5–7]. Premetamorphic frogs, or tadpoles, have a developed retina and possess the ability to protect their RGCs and promote axon regeneration following even complete transection of the optic nerve [8, 9]. In fact, tadpole RGCs regrow their axons from the site of injury and re-establish the optic nerve connection to the visual cortex, restoring functional vision [8]. An important advantage of tadpoles is their transparency, which allows studying all stages of optic nerve regeneration. However, while optic nerve regeneration in tadpoles is an indisputable fact, detailed analysis of the dynamics of this process and understanding of the mechanisms involved are rather limited. Having a standard, simple, and reproducible tadpole model can advance this understanding.

The small size of the tadpole makes optic nerve crush an impractical and imprecise technique with highly variable outcomes and highly mortality rates. Meanwhile, axotomy in the tadpole requires skilled microsurgical technique and similarly suffers from significant operator variability. Survivability and reproducibility are major obstacles even with the use of micromanipulators, stereo-dissecting microscopes, and fine tools. All these issues lead to highly variable data sets, limiting the power of conclusive results. To improve upon this, we developed a surgical model in *X. laevis* tadpoles using a laser microdissection platform that allows for fast and consistent optic nerve transections at a desired landmark every time. Using this model, we established the spatial and temporal dynamics of axon regeneration following optic nerve transection and evaluated the success of this regeneration in restoring visual function.

## Results

### Novel laser model allows us to perform accurate and reproducible optic nerve transection

**Figure 1:**
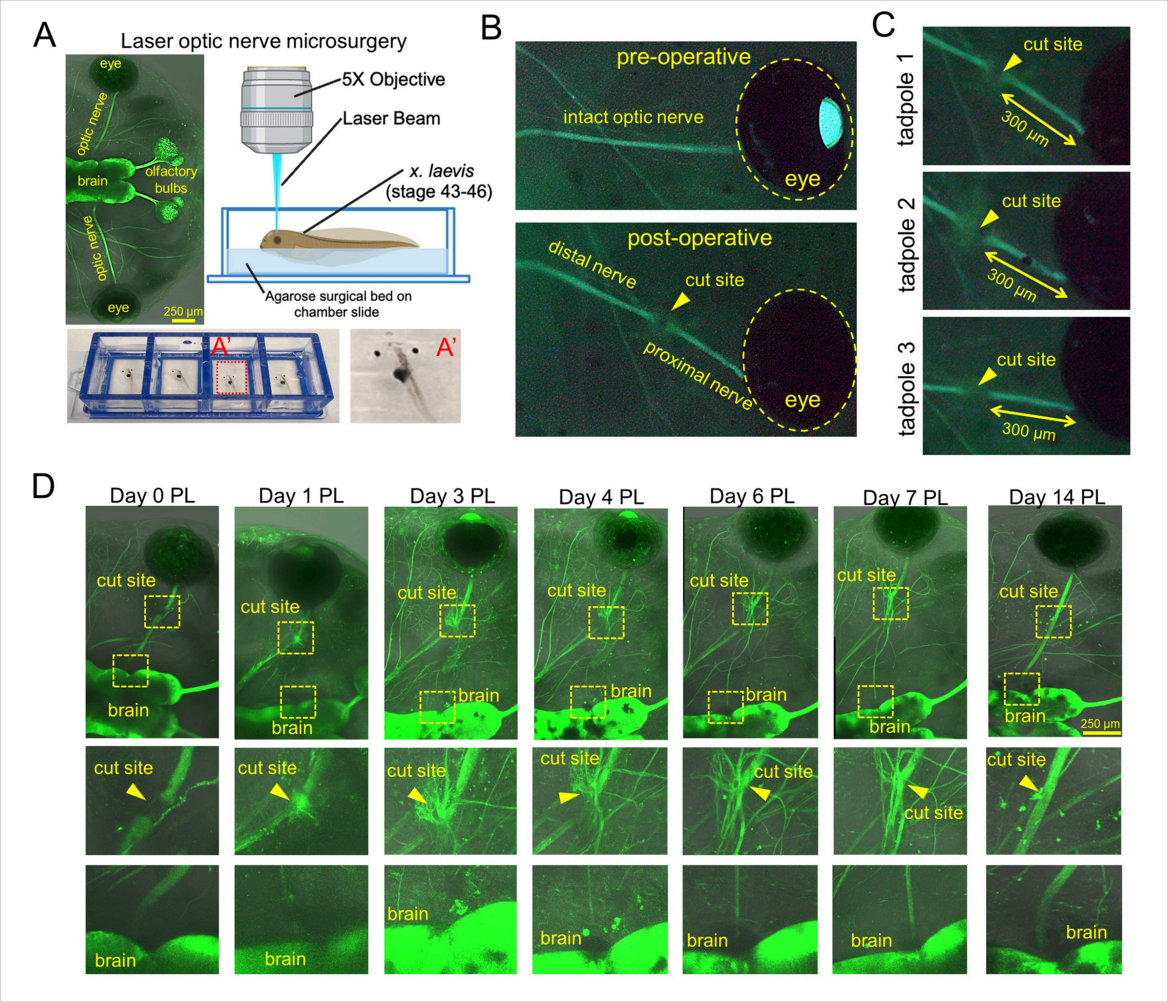
Novel laser model allows for accurate and reproducible optic nerve transections. **A)** Leica LMD7 laser microdissection experimental setup with Xla.Tg(tubb2b:mapt-GFP)^Amaya^ transgenic *X. laevis* line. **A’)** Tadpoles are anesthetized and loaded onto small surgical beds made of 4% agarose inside a chamber slide, so they are flat with their dorsal side up. **B)** Pre-operative and post-operative views of the endogenously labelled optic nerve in the GFP fluorescent channel. The gap in fluorescence in the post-operative image indicts the nerve was properly transected. **C)** Representative images of the precision and reproducibility of this model in 3 tadpoles with their nerves cut at the same distance from the eye. **D)** Longitudinal imaging of a tadpole after laser optic nerve transection. Axons distal to the injury site degenerate from day 0–2 post laser (PL), followed be several axons sprouting from the proximal cut end in all directions from the injury site by day 3 PL. Regrowing axons quickly reach the brain after which this path becomes strengthened. After 14 days, the optic nerve is virtually indistinguishable from the naïve (uninjured) optic nerve morphology.

To develop a new surgical model, we used the Leica LMD7 laser microdissection platform and Xla.Tg (tubb2b:mapt-GFP)^Amaya^ transgenic *X. laevis* line to visualize the optic nerve cut and the subsequent axonal regrowth. *X. laevis* tadpoles were used at developmental stages 43–46 because at these stages the tadpoles are transparent and suitable for live-fluorescent imaging (Fig. 1A). Tadpoles were anesthetized and loaded onto custom surgical beds (Fig. 1A’). The left optic nerve of each tadpole was transected with a 349 nm UV cutting laser at 5× magnification, 300 μm distal to the retina (using the “measure” tool in the Leica LMD7 software). The cut can be confirmed by a gap in the fluorescent optic nerve visualization (Fig. 1B, 1C). The tadpoles were immediately removed, recovered in 1X MMR media, and used in later experiments. Our survival rate with this model is 97.3% over many experiments, and nerves can be cut at precisely the same distance (Fig. 1C). Following our novel laser transection injury, we observed with longitudinal confocal imaging that axons distal to the injury site degenerate from day 0–3 post laser (PL), followed by regeneration of the optic nerve from the globe-proximal cut end, which is observed from the third day after optic nerve transection (Fig. 1D). After 14 days, the regrown optic nerve is anatomically and morphologically indistinguishable from the naïve (uninjured) optic nerve (Fig. 1D).

#### Pre-existing RGCs do not die and new RGCs are not generated from retinal progenitors after optic nerve transection

**Figure 2:**
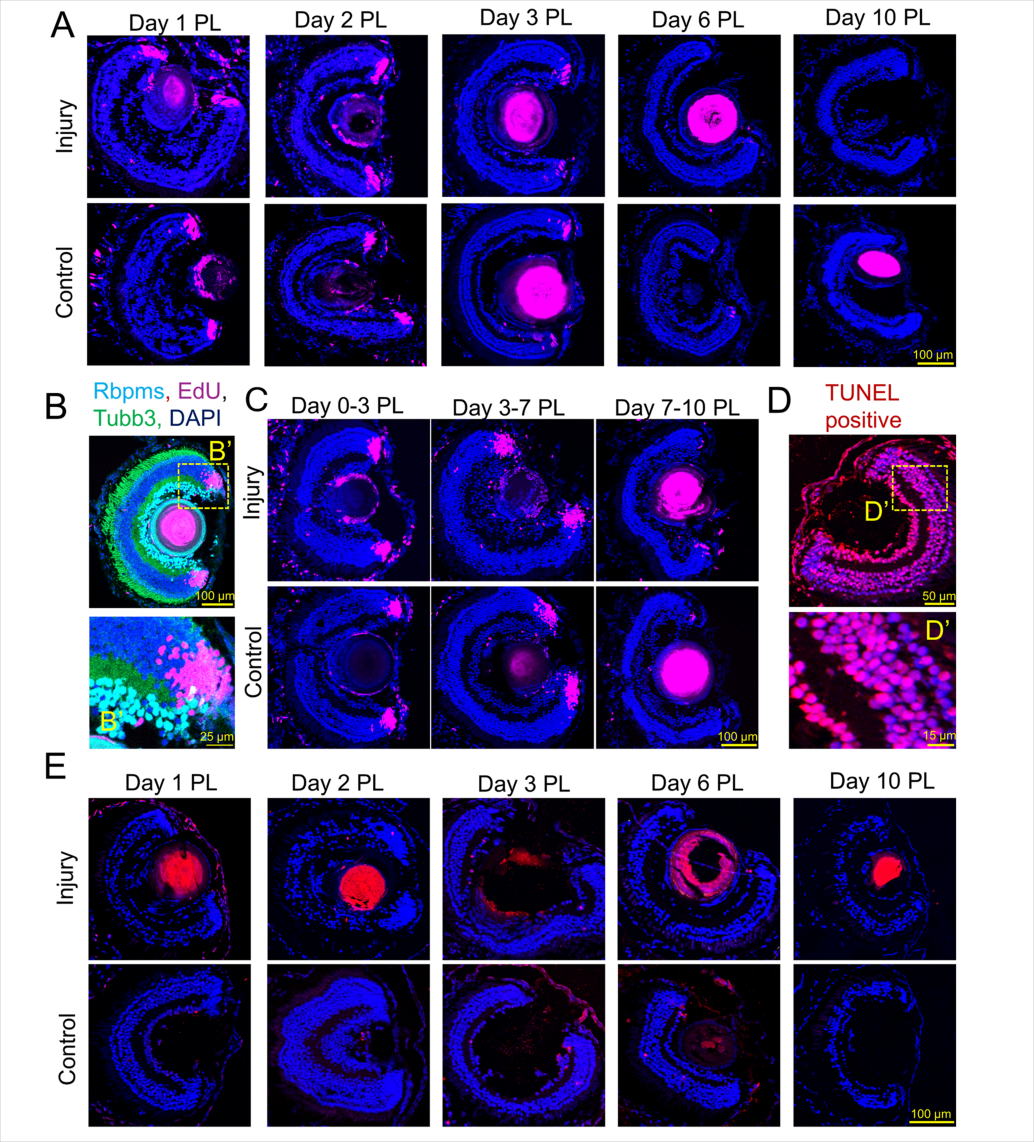
Pre-existing RGCs do not die and new RGCs are not generated from retinal progenitors after optic nerve transection. **A)** Control and experimental retinas at 1, 2, 3, 6, and 10 days post laser injury. EDU labeled cells are in magenta, cell nuclei are labelled in blue (DAPI). Tadpoles swam for 4 hours (pulse) in EdU-containing tadpole buffer (1X MMR), after which the retinas were collected for analysis of proliferating cells. **B)** Representative image of RGC layer in the retina labelled in cyan (RBPMS), Endogenous GFP (Tubb3), EDU in magenta and DAPI in blue. **B’)** close up of cells in the ciliary marginal zone in the retina. **C)** 3 day cumulative EDU labelling of cells. Tadpoles swam from day 0–3, 3–7, or 7–10 PL. D) TUNEL positive control. TUNEL positive cells are in red, cell nuclei are labelled in blue (DAPI). DNA strand breaks were induced with DNase I. **D’)** Close up showing almost every cell nucleus is positive for TUNEL staining. **E)** TUNEL control and experimental retinas at 1, 2, 3, 6, and 10 days post laser injury.

It is known that damage to axons can lead to RGC death, and that dying RGCs in amphibian retinas can trigger RGC differentiation from progenitors for replacement[10–15]. Thus, we explored whether in our model damage to axons leads to death of pre-existing RGCs, and if axonal regeneration is the result of sprouting of axons from newly differentiated RGCs. Since amphibian Müller glia and RPE undergo a reprogramming process and generate a pool of proliferating progenitor cells, we examined these cell types as a potential source of newborn RGCs after optic nerve injury in the model. To this end, the left optic nerves were cut (the right optic nerves were used as controls). Before collecting control and experimental retinas 1, 2, 3, 6, and 10 days after injury, the tadpoles swam for 4 hours (pulse) in EdU-containing tadpole buffer (1X MMR). The retinas were then collected for analysis of proliferating cells. After 1, 2, and 3 days after injury, there was staining for EdU in the ciliary marginal zone, the region of the tadpole retina which is responsible for normal retinal growth, which was equal in the control as well as the injured retina (n = 4, Fig. 2A, 2B). We found no EdU staining in the ciliary marginal zone in experimental and control tadpoles 10 days after damage (Fig. 2A). We also did not detect any staining in those areas of the experimental and control retinas where Müller glia and RPE are located (Fig. 2A). Since we found dividing cells only in the ciliary marginal zone, progenitors from this zone could be responsible for the appearance of newborn RGCs (Fig. 2B). To test this hypothesis, we used the following design. The optic nerve was cut and after 0, 3 or 7 days the tadpoles were placed in EdU- containing tadpole buffer for 3 days. We expected that such a period would be enough for cells differentiated from progenitors to take their place in the ganglion cell layer of the retina. We found only a small number of EdU-positive newborn cells in the ganglion cell layer, which did not differ between experimental and control retinas (n = 4, Fig. 2C). Next, we examine cell death in retinal slices using a TUNEL assay. To this end, the left optic nerves were cut, and the retinas were collected after 1, 2, 3, 6, and 10 days. The right optic nerves were used as controls. We also used TUNEL-positive controls (Fig. 2D). We found only a small number of TUNEL-positive cells (maximum 3 cells per retinae and sometimes no cells at all, n = 4) in both experimental and control retinas (Fig. 2E).

#### RGC axons sprout in all directions during an early stage of optic nerve regeneration, but the plasticity and mobility of RGC axons allows them to restore a full-fledged optic nerve at a late stage

**Figure 3:**
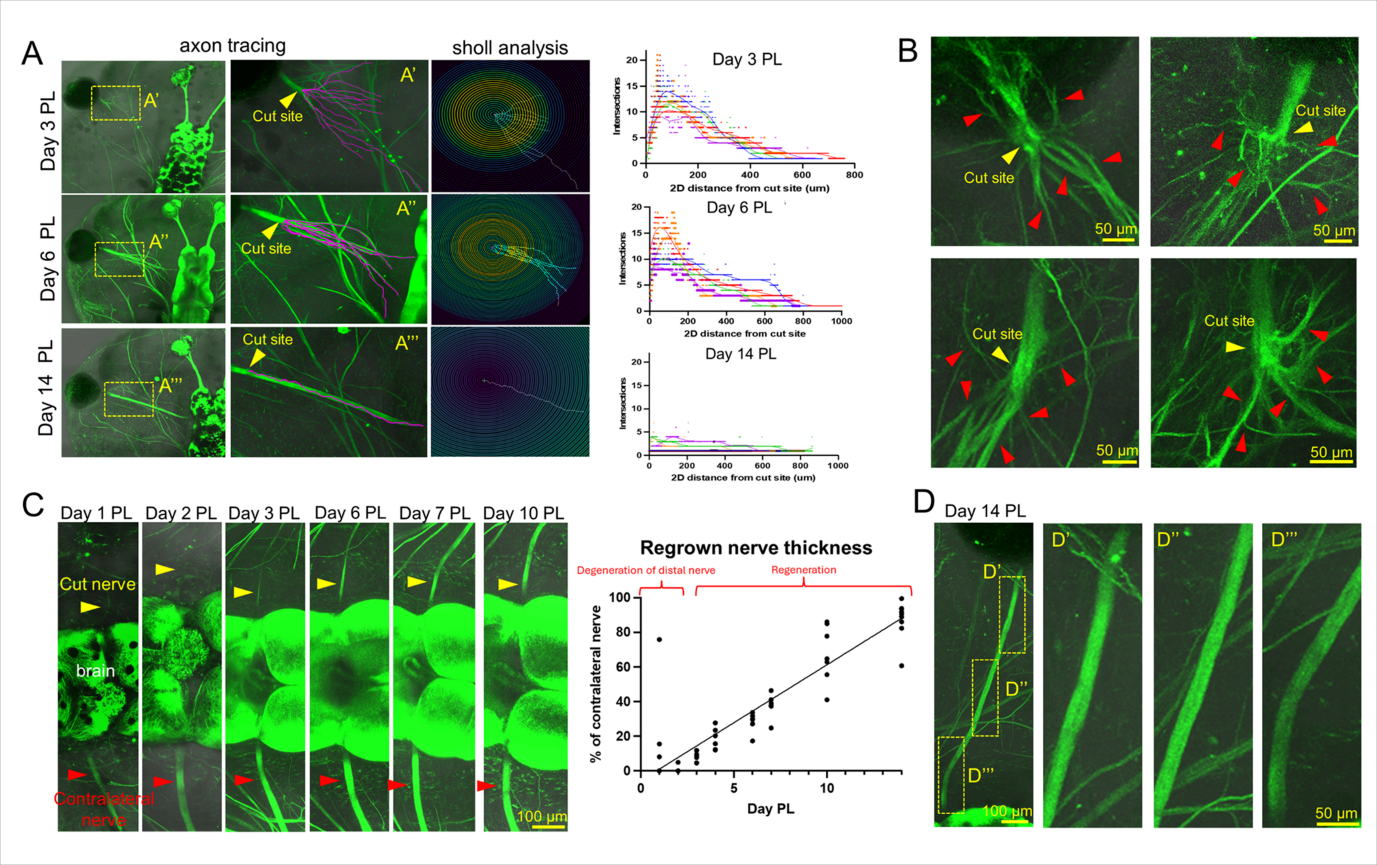
RGC axons sprout in all directions during an early stage of optic nerve regeneration, but the plasticity and mobility of RGC axons allows them to restore a full-fledged optic nerve at a late stage. **A)** Sholl analysis of axon patterns at 3, 6, and 14 days PL. **B)** Representative images of early stage axons which branch in all directions from the cut site. **C)** Quantification of regrowing nerve thickness at the point where it meets the brain compared to the contralateral nerve over time. **D)** Representative images of a late stage highly organized, regrown nerve.

We were unsure if the presence of a chemoattractant gradient could still be expected in our tadpoles at this stage of development. If a chemoattractant gradient were present, then the RGC axons should grow in a certain direction and not in all possible directions. To this end, we cut the optic nerve and observe how it regenerates in a time-dependent manner. To examine the patterns of axon growth and remodeling, we conducted Sholl analysis at 3, 6, and 14 days after injury (Fig. 3A). Axons were semi-automatically traced with the auto-snapping feature in Neuroanatomy/SNT plugin and then the Sholl analysis plugin was used to assess branching complexity by measuring the frequency at which the traced axons intersect concentric circles spaced at regular intervals from the origin (in our case, from the cut site). At 100 μm from the cut site, the number of intersections at day 3 was 11 ± 1 (n = 5 regrowing nerves), for day 6 it was 12 ± 1 (n = 5), and for day 14 it was at 2 ± 1 (n = 10). However, while the shape at day 3 and 6 after injury indicates many axons branching out from near the cut site, there are fewer branch intersection points the farther out from the cut site you go (Fig. 3A). We also noticed that there are many axons during these periods of time that form loops or randomly change their direction of movement (Fig. 3B). All these data indicate that a chemoattractant gradient is not present during an early stage of optic nerve regeneration. By day 14, however, there is only one line and the initial peak of intersections is gone (Fig. 3A). To better understand the process of formation of a full-fledged optic nerve after injury, we quantified the thickness of the regrowing nerve where it meets the brain compared to the contralateral nerve (Fig. 3C). At day 1 after injury, the percentage of the contralateral nerve was 55 ± 35% (n = 3, a large variation was observed in the sizes of the degenerating distal segment), and this was observed to be the degenerating distal segment. At day 2 the average was 1 ± 1% (n = 5) and the average of day 3 was 8 ± 3% (n = 6). At day 4 the average was 19 ± 6% (n = 6), and at day 6 the average was 28 ± 6% (n = 6). At day 10 the average was 70 ± 17% (n = 8). At day 14 the average was 87 ± 11% of the contralateral (n = 10). One would expect that in the absence of a gradient at a later stage, the emerging new optic nerve would be less organized. However, our data indicate that the new optic nerve does not differ from the control (contralateral) optic nerve at 14 days after injury (Fig. 3D). These results suggest that at a later stage, attraction occurs from axons that have established contact with the brain. This attraction may be caused by the secretion of chemoattractants from these axons and the formation of a gradient. This may also be caused by the fact that the axons which do not make it to the correct place in the brain are either rerouted or trimmed back and regrown.

#### Regrown nerve resembles the contralateral uninjured nerve in 3D spatial positioning, resulting in functional vision restoration

**Figure 4:**
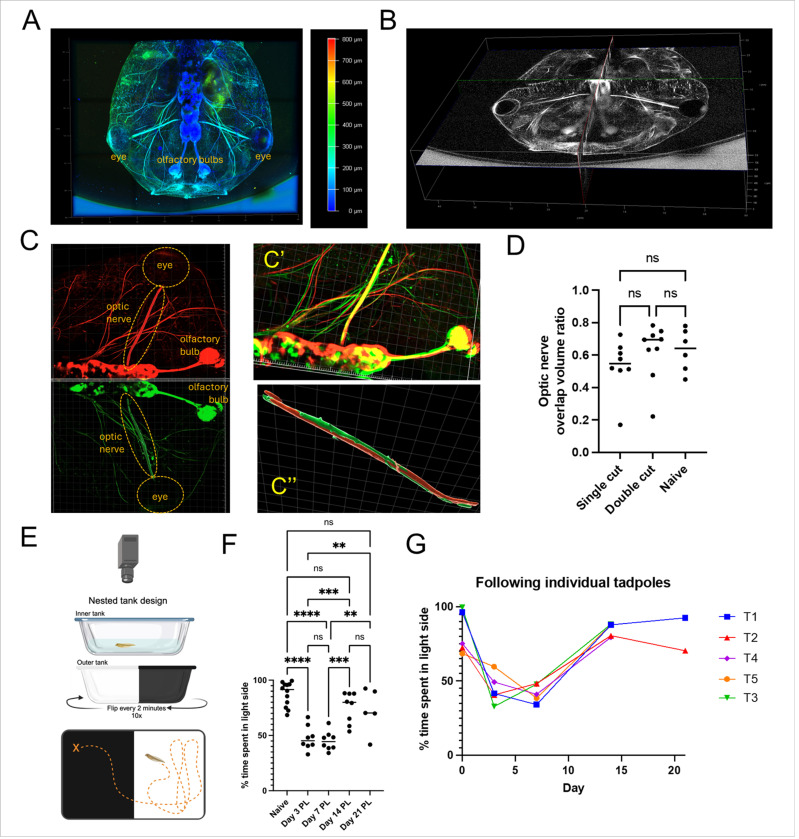
Regrown nerve resembles the contralateral uninjured nerve in 3D spatial positioning, resulting in functional vision restoration. **A)** Depth-coded imaging at day 14 PL (left side regrown). **B)** Orthogonal plane view with nerves intersecting the brain at day 14 PL. **C)** Overview of 3D volume colocalization analysis using Imaris image analysis software. Regrown nerve side is labelled in green and contralateral nerve side is pseudo-colored red. **C’)** The two halves are overlayed on the mirror plane in 3D space **C’)** Each nerve is made into a “surface” from which the overlap volume ratio can be calculated. **D)** Optic nerve overlap volume ratio results. There were no statistical differences at day 14 between single cut, double cut or naïve cohorts. **E)** Tadpole behavioral vision test setup. **F)** Quantification of percentage of time tadpoles swam in the light side before injury (naïve) and at Day 3, 7, 14 and 21 PL. **G)** Results of following the same individual tadpoles across these timepoints. T1=tadpole 1.

**Figure 5:**
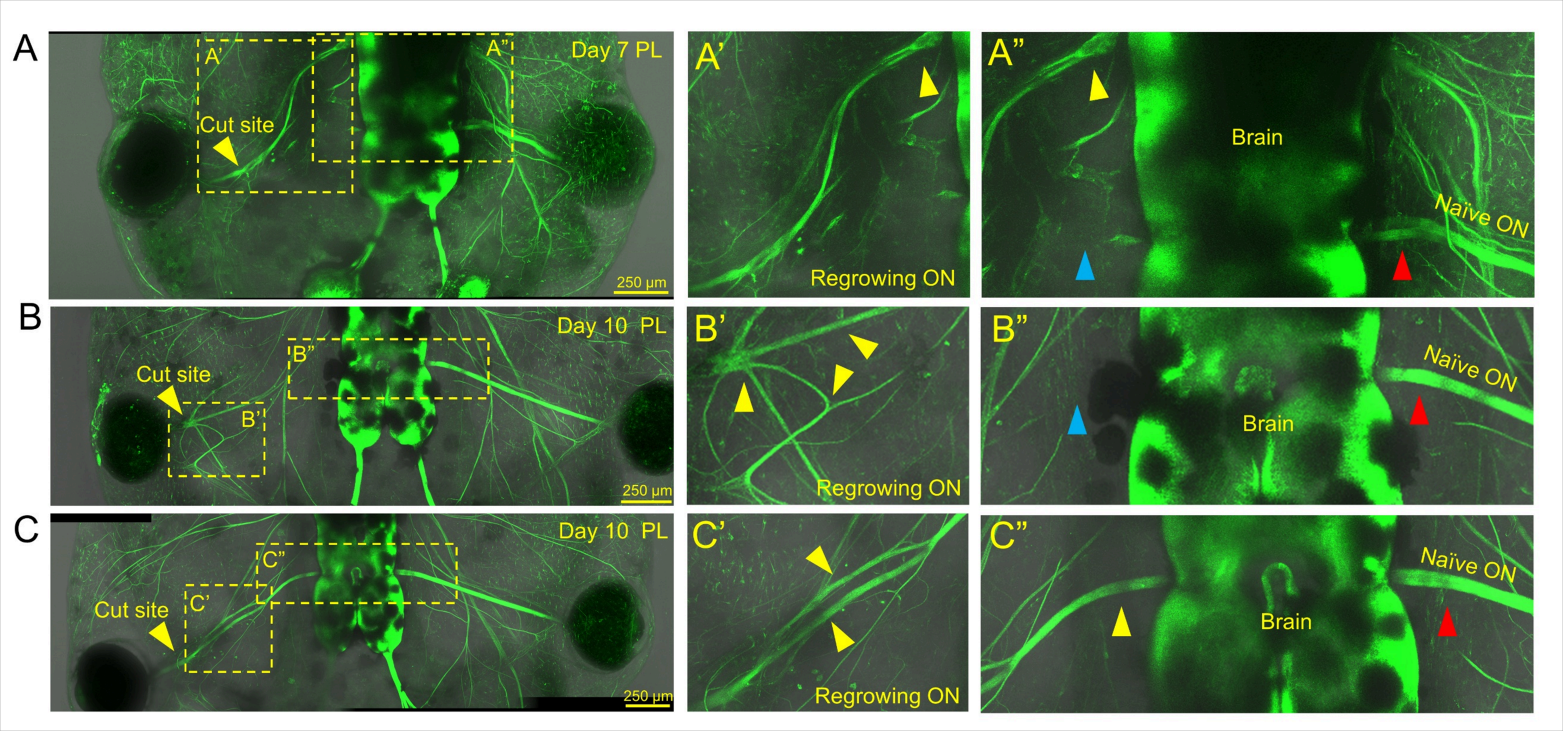
Three examples of the rare cases in which the axon pathfinding signals were mixed up and regrew to the wrong place, or did not resemble the organization of the contralateral nerve. **A)** An abnormally regrown day 7 PL tadpole which was lasered on the left side is shown. The regrowing optic nerve seems to have been rerouted to rejoin another nerve which is indicated by the yellow arrow (**A’**). The nerve has not regrown to the same place as the contralateral on the brain (**A”**). The correct location is indicated by the blue arrow while the regrown nerve is indicated by the yellow arrow (**A”**). **B)** A second case where a Day 10 PL tadpole was lasered on the left side and did not regrow normally. This tadpole seems to have strengthened the path back towards another nerve, and also several of the other axon branching paths at once (indicated with yellow arrows) (**B’**). However, none of the axon paths made it to the correct place on the brain compared to the contralateral (indicated by the blue arrow **B”**). **C)** The third case of a Day 10 PL tadpole has a partially divided regrown nerve compared to its contralateral uninjured ON. Here it seems like two axon paths may have made it to the correct part of the brain and were strengthened, resulting in a portion of the ON which is doubled (indicated with yellow arrows) (**C’**). Despite the two axon paths being strengthened and the resulting double portion of ON, the ON eventually does reorganize and regrow to the correct side of the brain as the contralateral (**C”**). Red arrows indicate the naïve contralateral nerve.

After observing consistent regrowth and remodeling of axons from pre-existing RGCs, we wanted to see if this robust optic nerve regeneration with our model resulted in useful vision. First, we checked if the regrown nerve eventually resembles the contralateral nerve in 3D spatial positioning. We performed depth-coded imaging and noticed that at 14 days, when the nerve most consistently resembles the contralateral, the depth of the regrown nerve was approximately the same and the point at which the nerve intersects with the brain was also in the same orthogonal plane (Fig. 4A, 4B). We designed an analysis to observe volume colocalization with the contralateral nerve overlayed on the mirror plane in 3D space using Imaris image analysis software (Fig. 4C). This was not a flawless system, because animals may not be biologically perfectly symmetrical with their left and right side, but since the tadpole is rapidly growing, this internal control was better than a pre-injury and post-injury overlay analysis. We used a naïve age-matched cohort to examine how much volume ratio overlap is typical in the left and right optic nerves with this imaging method, and if there were any differences when either one or both nerves are cut and regrown. There were no statistical differences at day 14 between single cut, double cut or naïve cohorts (Fig. 4D). These results indicate that even when both optic nerves are cut and there is no chemoattractant gradient early on, RGC axons have internal information that allows them to establish contact with the correct part of the brain. This statement is confirmed by our behavioral tests, according to which tadpoles practically restore vision on the 14th day after optic nerve injury (Fig. 4E–4G). We took advantage of the fact that sighted *X. laevis* tadpoles prefer a white background when given the choice between a white and black background (Fig. 4E) [16]. For all behavioral tests, we performed bilateral optic nerve transections to ensure complete blindness. We recorded swimming videos from naïve, as well as 3, 7, 14 and 21 days after injury. The naïve, uninjured tadpoles showed a clear preference for the white side of the tank (Fig. 4F). They spent an average of 88 ± 3% (n = 12) of the time on the light side of the tank. At day 3 and day 7 after injury, tadpoles exhibited no preference and swam back and forth across the black and white sides of the tank relatively equally (day 3: 48 ± 4%, n = 8; day 7: 45 ± 3%, n = 8; Fig. 4F, 4G). At day 14 and 21, however, the tadpoles again showed a preference for the white side of the tank (day 14: 75 ± 5%, n = 8; day 21 73 ± 9%, n = 5). The naïve percentage was significantly different (p < 0.0001) from both day 3 and day 7 post injury. However naive vs. day 14 and naïve vs day 21 were not significantly different. This is consistent with tadpoles losing vision after double injury, being blind at day 3 and day 7 and having regained vision by day 14 and day 21. Considering these results and the data presented above, we can propose the following model. Injury to the optic nerve results in the sprouting of axons from pre-existing RGCs in all directions. The RGC axons, upon reaching the brain, can recognize whether this part is correct or incorrect. If the axons reach the correct part of the brain, they strengthen contact with it and possibly create a gradient of attracting other axons. If the axons reach the wrong part of the brain, they are either rerouted or trimmed back and regrown. There were a few exceptions to this rule, and we have noticed on occasion axons which grew to the wrong location and strengthened contact to the incorrect location, such as towards another nerve, but these cases were very rare (Fig. 5).

### The optic nerve transection has a slight effect on the retina at the molecular level

**Figure 6:**
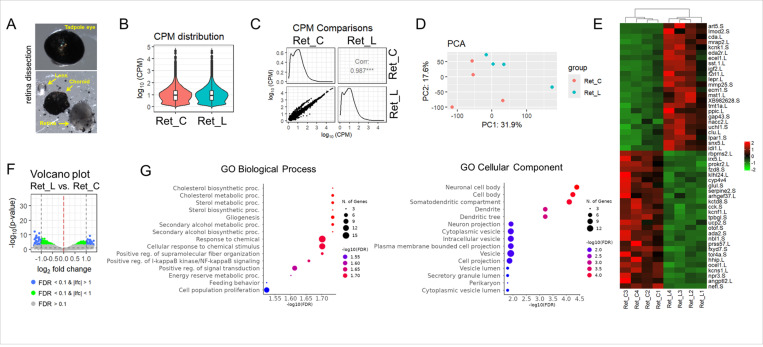
Cutting the optic nerve with a laser has a slight effect on gene expression in the retina at 3 days PL. **A)** Retinas from experimental and control eyes were collected for RNA isolation three days after optic nerve transection. **B)** RNA-seq data indicate that counts per million (CPM) distributions for control (Ret_C) and experimental (Ret_L) retinas do not differ significantly 3 days after injury. **C)** To demonstrate the correlation (Corr) between gene expression in Ret_C vs. Ret_L retinas, scatter plots were utilized **D)** The principal component analysis (PCA) shows a slight difference between control and experimental retinas. **E)**The heatmap shows genes whose expression differs greatly in Ret_C vs. Ret_L retinas. **F)** The volcano plot shows that the number of genes whose expression was statistically significantly (FDR<0.1) decreased or increased |lfc| >1 in experimental and control retinas was small. **G)** Subtle changes in gene expression are reflected in the low representation of significant biological processes or cellular components that may play a role in retinas with injured optic nerves.

Our morphological observations of proliferation and death of cells in the retina following optic nerve transection indicated the absence of significant changes compared to control retinas. However, how do these observations fit with changes in the retina at the molecular level? To answer this question, we performed RNA-seq analysis of the control and experimental retinas 3 days after the optic nerve transection. We chose this time point because at 3 days post optic nerve transection we observed significant axonal sprouting, which would be reflected in changes in expression in the retina. Due to the small quantity of material, we pooled 8 retinas to prepare one RNA sample (Fig. 6A). In total, we prepared 4 experimental and 4 control (retinas from the contralateral eyes) RNA samples that were used in the RNA-seq analysis. Since the retina contains many different cell types, we used deep sequencing to detect changes in gene expression in small populations of retinal cell types, including RGCs. To this end, 44,930,137 ± 2,304,700 fragments were sequenced on average per library among which 40,233,947 ± 2,096,169 fragments were uniquely mapped to the mouse genome. Our results indicate that counts per million (CPM) distributions do not differ between experimental and control retinas (Fig. 6B). While the value of the correlation coefficient (0.987), principal component analysis (PCA), heatmap and volcano plots show differences in expression levels between experimental and control retinas, the number of genes that showed a two-fold change in expression was small (Fig. 6C–6F, Supplementary Data S1). The number of genes whose expression was twice as high (log2 fold change [log2FC] ≥ 1) in the experimental retinas compared to the controls was 44 (FDR < 0.1); the number of genes whose expression was reduced more than twofold (log2FC ≤ − 1) in the experimental retinas compared to the control retinas was 38 (Supplementary Data S1). The gene ontology (GO) enrichment analysis revealed that genes with increased expression are involved in basic metabolic processes or in processes associated with the activity of neurons and glial cells (Fig. 6G, Supplementary Data S1). Among the genes with increased expression, gap43 (log2FC = 1.43, FDR = 0.014) and uchl1 (log2FC = 1.32, FDR = 0.073) are worth noting, the role of which in the process of axon regeneration after damage to the optic nerve has already been shown (Fig. 6E, Supplementary Data S1)[9]. It is interesting to note that the expression of clu (clusterin), the activity of which is characteristic of reactive astrocytes and Muller glia, was increased (log2FC = 1.25, FDR = 0.095). Meanwhile, Muller glia marker glul (glutamine synthetase) expression was reduced (log2FC=−1.23, FDR = 0.029). We also noticed reduced expression of a RGC marker rbpms2 (log2FC=−1.11, FDR = 0.014). However, we were unable to identify significant processes in which genes with reduced expression are involved. It should also be noted that we did not detect activity of signaling cascades associated with inflammation and cell death. Overall, our results could be described as that regeneration of RGC axons occurs in the resting state of the retina.

#### There is no significant inflammatory response in the optic nerve after injury, but the activity of signaling cascades involved in cell proliferation is increased

**Figure 7:**
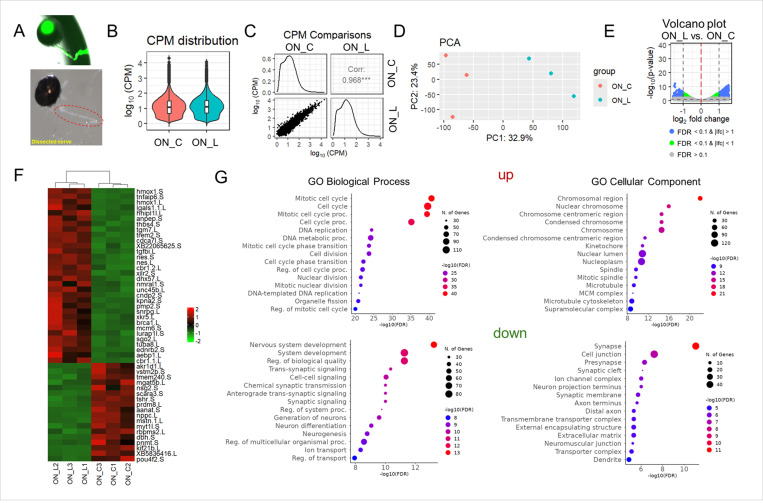
Optic nerve transection results in significant changes in gene expression at the lesion site at 3 days PL. **A)** Three days after injury, optic nerves were collected for RNA extraction and RNA-seq library preparation. **B)** RNA-seq library analysis indicates that CPM distributions do not differ between control (ON_C) and injured (ON_L) optic nerves. **(C-E)** Scatter plot matrix (**C**), PCA (**D**), and volcano plot (**E**) show significant differences between control and injured optic nerves at the gene expression level. **F**) Top significantly up- and down-regulated genes are shown in the heatmap. **G)** The gene ontology (GO) enrichment analysis shows that many genes whose expression is increased in injured optic nerves are involved in the cell cycle, while genes whose expression is decreased are involved in synaptogenesis.

The lack of significant changes in gene expression in the experimental retinas compared to control retinas prompted us to examine changes in gene expression in the optic nerves at the same time point (3 days post injury). To this end, tadpole nerves were lasered on the left side and 3 days later (the time of axon branching) left (experimental) and right (control) optic nerves were dissected. Due to the very low amount of RNA in nerves, we pooled 15 nerves per sample, 3 samples per condition - either lasered or control (Fig. 7A). These samples were used for RNA-seq analysis. While CPM distributions did not differ between experimental and control optic nerves, we found a significant difference in gene expression between experimental and control optic nerves as inferred from the value of the correlation coefficient (0.968), principal component analysis (PCA), and volcano plot (Fig. 7B-7E). We found that the expression of 457 genes was two-fold or higher in experimental optic nerves compared to control optic nerves, while the expression of 276 genes was two-fold or lower (FDR < 0.1, Fig. 7F, Supplementary Data S1). The lists of these genes were used in the GO enrichment analysis. We found that a significant number of genes upregulated in damaged optic nerves were involved in cell proliferation (Fig. 7G, Supplementary Data S2). This result may be explained by increased expression of genes involved in gliogenesis (e.g., GO:0014013 regulation of gliogenesis [FDR = 0.009]), which may be associated with the restoration of the optic nerve structure. Genes whose expression is reduced are mainly involved in synaptogenesis (Fig. 7G, Supplementary Data S2). The reduced expression of these genes may be explained in part by the fact that new axons have not yet established contact with the brain at this time point (3 days post injury). It was surprising to us that among the biological processes with a high enrichment FDR, there were none that were involved in the inflammatory response. Several processes involved in the inflammatory response can be found with low FDR (e.g., GO:0006954 inflammatory response [FDR = 0.011], GO:0002534 cytokine production involved in inflammatory response [FDR = 0.025], GO:0050728 negative regulation of inflammatory response [FDR = 0.035], Supplementary Data S2). However, the genes involved in these processes and having increased expression in the damaged optic nerves play a supporting rather than a key role (Supplementary Data S2). The absence of a toxic inflammatory response in the optic nerve after injury likely promotes axonal regeneration.

## Discussion

In this study, we present a novel optic nerve injury model using GFP β-tubulin-labelled transgenic *X. laevis* tadpoles and a laser microdissection platform that can be used to study robust RGC axon regeneration post-optic nerve injury in a very short timeframe. We found that optic nerve transection does not result in the death and *de novo* differentiation of RGCs from progenitors, but rather, that the regenerated optic nerve is formed entirely by the regrowing axons of pre-existing RGCs. Our data indicate the absence of a chemoattractant gradient at the early stage of optic nerve regeneration, which leads to defasciculated axonal sprouting in random directions. Our findings suggest that RGC axons which reach the correct visual centers of the brain can then attract new axons, which leads to the rapid formation of an optic nerve indistinguishable from the naive one. All these processes lead to the restoration of vision in tadpoles even if both of their optic nerves are transected. Optic nerve transection does not result in any significant changes in the retina at the morphological or molecular level. In the injured optic nerve, we observed activation of signaling cascades responsible for cell proliferation, which may be associated with other cellular components in the optic nerve and the restoration of the optic nerve structure.

Our findings align with previous studies on axonal regeneration in teleosts and amphibians. In our new tadpole model of nerve injury, we observed similar stages of axonal degeneration and regrowth as was seen in the adult *X. laevis* frog, only in a considerably shorter timeframe [9]. It is interesting as well that in the frog crush model, the optic nerve sheath is preserved, which we predict to greatly influence dynamics of axonal regrowth. Still, regenerating RGC axons in fish and frog have been shown to make pathfinding errors and initially establish imprecise connections [9, 17, 18]. This was followed by an activity-dependent refinement process, reminiscent of the synaptic refinement seen during development in goldfish [19]. It should also be noted that in the fish and frog crush models axon regeneration was shown to be the result of preexisting RGCs which survive the injury rather than *de novo* RGCs [8, 9, 20]. Since the tadpole is still developing, one concern is that injury paradigms may be confused with developmental ones. We were very diligent in ensuring that age matched tadpoles were kept in the same conditions as controls, and use internal controls (ex. contralateral uninjured eye) when possible. Because optic nerve surgeries in tadpoles require highly skilled techniques and results may vary greatly, our novel laser model provides a more controlled environment which solves these issues.

The key problems preventing optic nerve regeneration in mammals are RGC death and the regenerative failure of the axons from surviving RGCs to reach the correct part of the brain and reforming the optic nerve [10, 11]. Since tadpoles can regenerate the optic nerve, we paid special attention to both of these processes in our laser model. The death of RGCs and the subsequent formation of the optic nerve from axons of newborn RGCs could explain differences with optic nerve formation in mammals. However, we did not find RGC death after optic nerve transection, and a new optic nerve is formed from sprouting axons of pre-existing RGCs after our laser transection injury. Thus, RGC differentiation from progenitors is not required for optic nerve regeneration in tadpoles, potentially minimizing the complexity of achieving optic nerve regeneration in mammals. We also found that optic nerve regeneration begins with a random process of axonal sprouting in arbitrary directions, indicating that there is no chemoattractant gradient in tadpoles at this stage. However, a gradient may arise from axons that have innervated the correct regions of the brain. If mammalian axons were also able to leverage the innervation of appropriate regions of the brain to establish a gradient for other RGC axons to reinforce a regenerating optic nerve, this would significantly advance the task of optic nerve regeneration in mammals. However, this would require avoiding the formation of a glial scar that typically impedes axonal sprouting and promoting a permissive environment for axon growth. This model could provide insights to the problem of eye transplantation.

The ability to heal in mammals is hindered by the injury response and the optic nerve possesses a severely limited capacity to repair following injury [21]. Many times, the secondary inflammatory and metabolic processes that follow initial damage to the nerve further progress and exacerbate the injury instead of facilitating recovery [22]. Growth-inhibitory and pro-inflammatory cellular debris impede axonal regeneration from the surviving RGCs and the connection to the brain critical for vision restoration. The retina and optic nerve are the most metabolically active tissues of the body, and among the earliest and most robust responses that occur after optic nerve injury in mammals are a pro-inflammatory response, mitochondrial dysfunction, oxidative damage, and corresponding overloads of antioxidant systems [23]. Eventually, this leads to permanent vision decline. Our data indicate these processes are not triggered in the xenopus retina or optic nerve at day 3 after cutting the optic nerve with a laser. We observed minimal changes at the molecular level in the retina of the injured optic nerve. Transection of the optic nerve had a greater impact at the molecular level. However, increased activity of cell cycle-related signaling cascades may be associated with glial cell differentiation and vascular sprouting, which is important for the restoration of the optic nerve structure. It is known that tissue damage can cause a strong inflammatory response and oxidative stress [12, 13, 24]. Therefore, there may also be an association with the reduced level of inflammatory response and oxidative damage and a consistent and minimal level of laser-induced damage to the optic nerve. This approach may have many advantages in future applications.

Tadpoles possess the ability to protect their neurons and promote axon regeneration following even complete transection of the optic nerve and are a much higher-throughput model than the adult frog. However, a major obstacle to working with small animal surgical models is the consistency and reproducibility of the procedures on each specimen, which yield highly variable datasets, are time-consuming and limit the power of conclusive results. The laser model described here allows us to bypass these obstacles and obtain important data on the mechanisms of optic nerve regeneration. Insights from this model may find its application in clinical practice, for example, in eye transplantation.

## Methods

### Animals

Premetamorphic Xla.Tg (tubb2b:mapt-GFP)Amaya transgenic *X. laevis* tadpoles, obtained from the Marine Biological Laboratory, Chicago, IL (https://www.mbl.edu), were used for all experiments. The tadpoles were maintained in 1X MMR at 19°C in a humidified incubator. Surgeries were performed at stage 43–46 [25]. Tadpoles were anesthetized by immersion in 1:3000 dilution of tricaine methanesulfonate (MS222, Sigma, St. Louis, MO) in normal amphibian media for 1 to 2 minutes. Animals were treated in accordance with the National Research Council’s Guide for the Care and Use of Laboratory Animals and the ARVO Statement for the Use of Animals in Ophthalmic and Vision Research. Animal procedures were approved by the Institutional Animal Care and Use Committee at the University of Miami.

### Laser optic nerve transection model

Tadpoles were anesthetized and loaded onto small surgical beds made of 4% agarose inside a chamber slide, with a small hole for the tadpole gut-region scooped out so that the tadpole is held evenly and flat on the agarose with its dorsal side up (Fig. 1A). This setup is very important because if the tadpole is too tilted, the laser will cut at a different angle and could cause more damage, or not transect the optic nerve properly. Anesthetized tadpoles were loaded onto LMD7 laser microdissection (Leica Microsystems, Exton, PA) specimen holder and kept moist. The left optic nerve of each tadpole was transected with a 349 nm UV cutting laser at 5× magnification, 300 μm distal to the retina (confirmed by using the “measure” tool). For behavioral tests, we cut both optic nerves. A line drawn across the optic nerve with the “draw” tool received four laser pulses (120 μJ/pulse) of 55 mW laser (pulse frequency = 2710), laser screw mode (step count = 2, step size = 5 μm, repeats = 2) to cut to the appropriate depth and transect the nerve. The cut can be confirmed by a gap in the fluorescent optic nerve visualization. Tadpoles were immediately removed and recovered in 1XMMR media. The detailed settings were as follows: Revolver position: 2, magnification: 5x, power: 50, aperture: 10, speed: 50, line spacing for draw + scan: 5, head current: 100%, pulse frequency: 2710, offset: 87, laser screw: step count: 2, step Nr.: 1, step size: 5 μm, repeats: 2 – and laser screw was applied between 3–4 times to ensure proper depth to cut nerve.

### TUNEL assay

We tested cell death in retinal slices using Click-iT Plus TUNEL Assay Kits for In Situ Apoptosis Detection (C10617, ThermoFisher) according to manufacturer’s instructions.

### EdU cell proliferation assay

We label dividing cells using EdU-Click kit (BCK-EDU647, MilliporeSigma) according to manufacturer’s instructions. To detect retinal progenitors activated by damage to the optic nerve, tadpoles swam for four hours (pulse) in 1XMMR containing EdU (1mM). After four hours, the tadpoles were immediately used in the analysis. To detect regenerated retinal cells after optic nerve injury, tadpoles were kept in 1XMMR containing EdU for an additional three days.

### Immunohistochemistry

Freshly dissected eyes were fixed in 4% MEMPFA (0.1 MOPS (pH 7.4), 2 mM EGTA, 1 mM MgSO4, 4% paraformaldehyde) overnight at 4°C, and infiltrated with 30% sucrose in phosphate-buffered saline (PBS) for 24 hours. The samples were then cryo-embedded in OCT, stored at − 80°C and cryo-sectioned at 14 μm thickness. Sections were permeabilized in 0.3% triton for 1 hour, and then blocked with 2% BSA + 0.15% tween + 5% donkey serum for 1 hour. Slides were left overnight in block and primary antibody (1:200) at 4°C. Then, slides were washed 3× 15–20 min each with 1x pbs and 0.15% tween. Secondary antibodies (1–1000 donkey anti-rabbit or donkey anti-mouse) were incubated at room temperature for 2 hours in PBS and 0.15% tween. Slides were washed 3× with 1× PBS and 0.15% tween with Hoechst (1:2000) for 20 min in first wash, and mounted with mounting media and coverslip.

Imaging was performed with Leica AOBS SP8 confocal microscope (Leica Microsystems, USA).

### Sholl analysis and optic nerve thickness measurements

Tadpoles were anesthetized and placed in a small drop of MS222 in media on a cover-glass bottom petri dish. Z-stack tile scan confocal microscopy images were acquired on a Leica AOBS SP8 (Leica Microsystems, Exton, PA). Confocal images of optic nerve axons were analyzed as previously described using the freely available Neuroanatomy/SNT Sholl analysis plugin available for FIJI/ImageJ (https://imagej.net/ij/). Axons were semi-automatically traced with the auto-snapping feature in Neuroanatomy/SNT plugin and then the Sholl analysis was used to assess branching complexity by measuring the frequency at which the traced axons intersect concentric circles spaced at regular intervals from the origin (in our case, from the cut site). We used a polynomial fit degree 11, semi-log Sholl decay, radius step size 0.2 μm. To study optic nerve thickness, confocal images were obtained before the injury and at day 1, 2, 3, 4, 6, 7, 10 and 14 PL and were analyzed in FIJI/ImageJ. The pixel to μm scale was set, and both the contralateral and regenerating nerves thickness were measured approximately 35 μm from where the nerve meets the brain. The regenerating nerve thickness was compared to the contralateral intact nerve thickness.

### Imaris image analysis

The concept was to overlay the contralateral control and regrown optic nerve sides of the tadpole head on the mirror plane and to do a colocalization analysis of the optic nerves in 3D space. We acquired 3D z-stack/tile scan images of the tadpole at 14 days PL, once the new optic nerve has regrown, and analyzed the images on the Imaris software (Oxford Instruments). For every animal, a normal stack and a flipped stack were prepared in FIJI (to mirror, z-stacks were opened in FIJI, Image was selected, then Transform, then Flip Horizontally) and saved as a new flipped stack. Then tif stacks were converted using the Imaris File Converter v10.1 to ims files and loaded into the Imaris software v10.1. Using the Crop 3D function, we cut the 3D stacks (both normal and mirrored) in half down the brain. For the mirrored image, the pseudo-color was changed to red and the normal image was kept green. We identified the regrown and contralateral control optic nerves as unique surfaces (or objects), and labeled them in different colors. Then we used the object-object statistics function to get the overlapped volume ratio of the two nerves and the z-coordinates of each.

### Functional vision behavioral test

For all behavioral assays, optic nerves were cut bilaterally. Behavioral tests were performed as previously published in JOVE [16], taking advantage of *X. laevis* preference for the white side of a white/black chamber when they have functional vision. Briefly, a single tadpole is placed in an inner clear tank that has 1XMMR buffer for tadpoles to swim freely in, and that tank is nested in an outer tank which is exactly half black and half white. Every 2 minutes, the inner tank is removed, the outer tank is flipped around so the white is where the black used to be and vice versa, and the inner tank replaced. This is done for a total of 10 times per tadpole (20 minutes total) per timepoint. Videos of individual tadpoles were recorded, and percent of time spent in the white side/total time swimming was quantified. This procedure was repeated at various time points post-injury (naïve, 3 days PL, 7 days PL, 14 days PL and 21 days PL), throughout the process of optic nerve regeneration.

### RNA purification, quality control and RNA-seq library preparation

RNA was isolated from retinas and optic nerves using RNeasy Plus Mini Kit (#74134, Qiagen, Hilden, Germany) according to manufacturer’s instructions [26]. RNA quantity and quality was determined by Qubit 4 Fluorometer and the NanoDrop One spectrophotometer (ThermoFisher Scientific, Waltham, MA, USA). To assess RNA integrity, 2100 Bioanalyzer Instrument (Agilent Technologies, Santa Clara, CA, USA) was used. The RNA samples with a RIN score of 8 or higher were used further. To prepare non-stranded RNA-seq libraries, we used mRNA-seq Lib Prep Kit for Illumina (RK20302, Abclonal, Woburn, MA, USA) according to manufacturer’s instructions. The RNA-seq libraries were sequenced from both ends on the Illumina Novaseq 6000 with a 2 × 150 paired end (PE) configuration. The FASTQ files generated in this study were uploaded to the BioProject database (https://www.ncbi.nlm.nih.gov/bioproject/) and are available under the accession number PRJNA1152608.

### RNA-seq data analysis

To align paired-end reads, we used STAR ultrafast universal RNA-seq aligner [27]. To create the STAR index for the reference genome, we used *X. laevis* v10.1 genome assembly. The HTseq package was employed to count reads that overlap each of the genes [28]. We used the edgeR R Bioconductor package to perform the differential gene expression analysis [29]. The ViDGER R Bioconductor package was used to visualize the results of the edgeR analysis. To perform principal component analysis (PCA) and generate heatmaps, we used ggplot2, ComplexHeatmap, and circlize R libraries as well as R functions. We used ShinyGO (ver. 0.80) for the gene ontology (GO) enrichment analysis [30].

### Statistical Analysis

All statistics were accomplished on Prism GraphPad 10.1.1. For experiments containing one variable, the unpaired Student’s t-test was applied. For experiments containing two or more variables, ANOVA was utilized. P values equal to or less than 0.05 were considered statistically significant. We always used positive and negative controls in our study. Generation and analysis of next-generation sequencing (NGS) data were performed in-house according to ENCODE standards and pipelines.

## Supplementary Material

Supplement 1

## Data Availability

The datasets generated and analyzed during the current study are available in the BioProject database (accession number PRJNA1152608) and in the article/Supplementary Data.
