# Supplementary material for: Novel laser model of optic nerve transection provides valuable insights about the dynamics of optic nerve regeneration
Source: Res Sq. 2024 Nov 2:rs.3.rs-5085599. Preprint. [Version 1] doi: 10.21203/rs.3.rs-5085599/v1 (PMC11581122; doi:10.21203/rs.3.rs-5085599/v1)
Supplement: Supplement 1 [file NIHPPRS5085599V1-supplement-1.pdf]

Supplementary Files

This is a list of supplementary files associated with this preprint. Click to download.

- SupplementaryDataLegends.docx
- SupplementaryDataS1.xlsx
- SupplementaryDataS2.xlsx
- SupplementaryMovieM1.mp4
